# Harnessing p57 Immunohistochemistry to Enhance Diagnostic Accuracy in Molar Pregnancies: An Insight

**DOI:** 10.7759/cureus.83716

**Published:** 2025-05-08

**Authors:** R S Kavyah, Shubha R Sangeetha, Shaista Choudhary, Abinaya Loganathan

**Affiliations:** 1 Pathology, Dr. B.R. Ambedkar Medical College & Hospital, Bangalore, IND

**Keywords:** complete hydatidiform mole, discordant, divergent, hydropic change, p57 immunohistochemistry, partial hydatidiform mole, products of conception

## Abstract

Introduction

Molar pregnancy (hydatidiform mole) is a premalignant form of gestational trophoblastic disease, occurring in approximately one in 1,000 pregnancies. It is characterized by abnormal swelling of chorionic villi and excessive trophoblastic proliferation. Accurate differentiation between complete hydatidiform moles (CHMs) and partial hydatidiform moles (PHMs) is crucial, as CHMs carry a higher risk of progressing to persistent trophoblastic disease or choriocarcinoma. p57 immunohistochemistry (IHC), regulated by the maternally expressed *CDKN1C* gene, is a key diagnostic tool due to its distinct expression patterns in different molar subtypes.

Methods

A prospective observational study was conducted over 18 months at Dr. B.R. Ambedkar Medical College & Hospital, in Bangalore, India, involving 25 cases: 15 clinically diagnosed molar pregnancies and 10 products of conception used as controls. Detailed clinical histories were recorded, followed by histopathological examination and p57 IHC analysis. All cases underwent statistical evaluation.

Results

Among the 25 cases, 12 (48%) were diagnosed as CHM, three (12%) as PHM, and 10 as non-molar controls. CHM cases showed negative p57 staining in villous cytotrophoblasts, whereas PHM cases exhibited weak to moderate nuclear positivity. Control cases demonstrated strong nuclear positivity. The use of p57 IHC showed a statistically significant correlation (p < 0.001) with accurate differentiation between complete and partial moles.

Conclusions

This study confirms that p57 IHC is a reliable and effective diagnostic tool for distinguishing between CHM and PHM. Its clear differential staining pattern enhances diagnostic precision, informs clinical decision-making, and minimizes the risk of misdiagnosis, supporting its routine implementation in the evaluation of molar pregnancies.

## Introduction

Hydatidiform mole is an abnormal form of gestation characterized by diffuse or focal hydropic changes and varying degrees of trophoblastic proliferation within the chorionic villi. It is the most common form of gestational trophoblastic disease (GTD) and is generally benign in nature [1]. These lesions can closely resemble non-molar abortions, various non-trophoblastic abnormalities, and developmental patterns observed in early normal placentation. Therefore, recognizing the morphological features of molar pregnancies is essential to distinguish them from these mimics [2].

Hydatidiform moles, along with invasive moles, choriocarcinomas, and placental site trophoblastic tumors, comprise the spectrum of GTD, with hydatidiform moles being the most frequent subtype [3]. Persistent GTD occurs in approximately 0.2-5% of cases of partial hydatidiform mole (PHM) and in 15-25% of complete hydatidiform mole (CHM) cases. In India, the incidence is estimated to be around one in 160 pregnancies [4]. Globally, hydatidiform mole is the most prevalent form of GTD, with an incidence of approximately one in 1,000 pregnancies, while in Asia, rates range from 0.8 to 3.0 per 1,000 pregnancies [5].

The pathological examination of products of conception (POC) remains the standard approach to differentiate among hydropic abortus (HA), PHM, and CHM. Due to their differing risks for clinical persistence and malignant progression, accurate classification and clinical correlation are essential. Histopathologic evaluation of tissue obtained via uterine curettage is considered the gold standard for diagnosing hydatidiform mole [6].

The p57 gene, located on chromosome 11p15.5, encodes a cyclin-dependent kinase inhibitor protein. It is an imprinted gene, expressed predominantly from the maternal allele, while the paternal allele is silenced. Its nuclear expression is detectable through immunohistochemistry (IHC). In normal tissue, nuclear staining for p57 is seen in villous cytotrophoblasts and villous stromal cells. Decidual tissue and extravillous/implantation site trophoblasts also exhibit positive staining and serve as internal controls. In cases of CHM, p57 IHC typically shows negative staining in villous cytotrophoblasts. This staining pattern, positive in villous stromal cells and decidua but negative in cytotrophoblasts, is crucial for distinguishing complete moles from partial molar pregnancies [7].

## Materials and methods

This prospective observational study was conducted in the Department of Pathology at Dr. B.R. Ambedkar Medical College & Hospital, in Bangalore, India, over an 18-month period, from March 2023 to September 2024. The primary objective was to assess the role of p57 IHC in identifying distinct staining patterns and evaluating its diagnostic utility in differentiating molar from non-molar gestations.

Study population

The study included a total of 25 cases, divided into two primary groups based on histopathological diagnosis: (1) molar pregnancy cases, comprising patients diagnosed with either CHM or PHM according to morphological criteria, and (2) non-molar abortions, consisting of patients whose POC showed no histopathological features indicative of molar pregnancy. A convenience sampling technique was used, wherein all eligible and available cases that met the inclusion criteria during the study period were included to enhance real-world data capture and relevance.

Clinical and histopathological data collection

Detailed clinical information was recorded for each patient, including maternal age, gravidity, parity, gestational age at presentation, and prior pregnancy outcomes. All tissue samples were fixed in 10% neutral buffered formalin for 24-48 hours and processed routinely.

Tissues were dehydrated through graded alcohols, cleared with xylene, and embedded in paraffin wax using an automated tissue processor. Sections measuring 3-5 µm were cut using a rotary microtome and mounted on clean, grease-free glass slides. These were stained with H&E following standard procedures.

Based on histopathological assessment, cases were classified as CHM, PHM, or non-molar (hydropic or spontaneous abortion).

IHC was performed on all cases to evaluate p57 expression - a gene maternally expressed and paternally imprinted, typically absent in CHM. Additional 3-5 µm sections were cut from paraffin blocks and mounted on poly-L-lysine-coated slides to ensure strong tissue adhesion. Heat-induced epitope retrieval was carried out using citrate buffer in a microwave or pressure cooker for 15-20 minutes. Slides were then incubated with a mouse monoclonal anti-p57 antibody (clone and dilution per manufacturer’s guidelines) for 30-60 minutes. Detection was performed using a biotin-streptavidin or polymer-based system, followed by chromogen development. Nuclear dot staining was interpreted as a positive result.

Evaluation of p57 expression

p57 expression was assessed in villous cytotrophoblasts, extravillous trophoblasts, and decidual stromal cells. CHM cases exhibited absent nuclear staining in villous cytotrophoblasts and stromal cells, while PHM and non-molar abortion cases showed positive nuclear staining. Positive staining in decidual tissue and intermediate trophoblasts served as internal controls to validate the IHC process.

Statistical analysis

Data were compiled and analyzed using descriptive statistics (mean, SD, and proportions). Comparative analysis among CHM, PHM, and non-molar gestations was conducted using the chi-square test or Fisher’s exact test for categorical variables, as appropriate. A p-value ≤ 0.05 was considered statistically significant.

## Results

A total of 25 cases were included in the present study. Demographic and clinical features, histopathological diagnoses, p57 IHC results, and their associations were evaluated.

**Figure 1:**
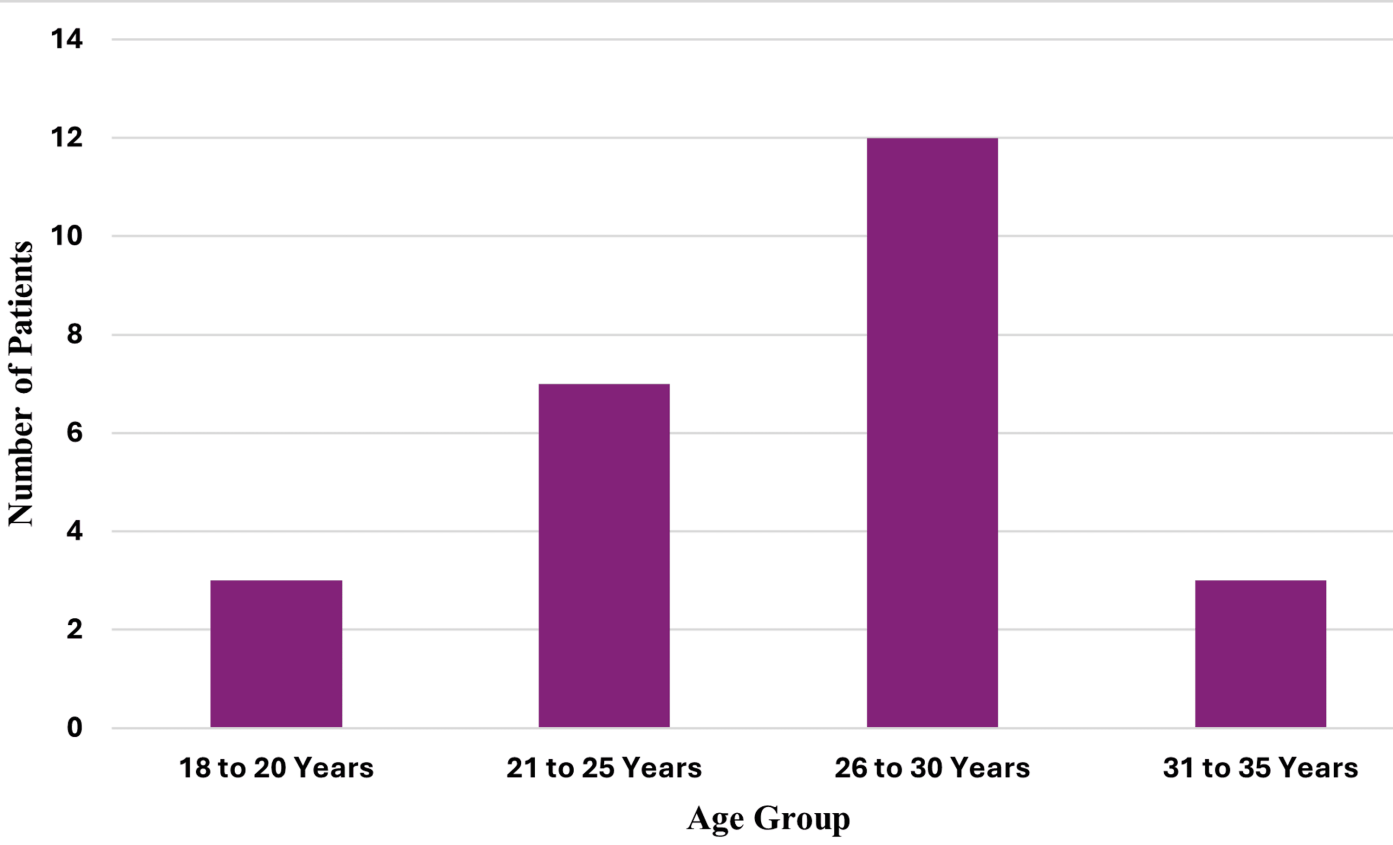
Age group segmentation

The age distribution of participants is illustrated in Figure 1. The mean age was 26.8 years, while the median age was 27 years. Most participants (48%, n = 12) were between 26 and 30 years old, followed by 28% (n = 7) aged 21-25 years. The age groups 18-20 years and 31-35 years each accounted for 12% (n = 3) of the participants.

Primigravida women constituted the largest proportion of participants at 52% (n = 13). Among the multigravida cases, 16% (n = 4) were gravida 2 (G2), 4% (n = 1) were gravida 3 (G3), 20% (n = 5) were gravida 4 (G4), and 8% (n = 2) were gravida 6 (G6).

A notable portion of participants (40%, n = 10) reported no clinical complaints. Anemia was the most frequently reported condition (32%, n = 8), followed by polycystic ovarian disease at 12% (n = 3), menorrhagia at 8% (n = 2), and both diabetes and seizure disorders at 4% (n = 1) each. A past history of molar pregnancy was present in 20% (n = 5), while 80% (n = 20) reported no such history.

Histopathological examination (HPE) findings revealed CHM in 48% (n = 12) of cases, PHM in 12% (n = 3), hydropic POC in 4% (n = 1), and non-molar POC in 36% (n = 9).

p57 immunohistochemical staining showed that 48% (n = 12) of cases exhibited expression in the 10-15% range. Other staining distributions included 15-30% and 30-50% (each 8%, n = 2), 50-70% (24%, n = 6), and >70% (12%, n = 3).

Inferential analysis

Association Between Age and Histopathological Diagnosis

CHM was most frequently observed in the 26-30 years age group (50%), followed by the 21-25 years age group (25%). However, no statistically significant association was found between age and HPE diagnosis (χ² = 3.274, p = 0.952) (Table 1).

**Table 1:** Association between HPE reports and age group CHM, complete hydatidiform mole; PHM, partial hydatidiform mole; POC, products of conception

Histopathology report	Age in years				Total	X²	p-value
	18-20	21-25	26-30	31-35		3.274	0.952
CHM	2 (16.6%)	3 (25%)	6 (50%)	1 (8.4%)	12		
Hydropic POC	0 (0%)	0 (0%)	1 (100%)	0 (0%)	1		
PHM	0 (0%)	1 (33.3%)	1 (33.3%)	1 (33.4%)	3		
PHM	1 (11.1%)	3 (33.3)	4 (44.5)	1 (11.1%)	9		

Association Between Obstetric Score and Histopathological Diagnosis

CHM was the most common histological finding, observed in 48% (n = 12) of cases, with primigravida women accounting for the majority (58.4%). The remaining CHM cases were distributed across G2, G4, and G6. PHM was found in 12% (n = 3) of cases, while POC accounted for 36% (n = 9) of cases. Only one woman with G3 obstetric status had hydropic POC (4%). A statistically significant association was found between obstetric score and HPE diagnosis (χ² = 30.016, p = 0.003). Among participants with a past history of molar pregnancy, CHM accounted for 33.4%, and PHM for 33.3%. However, the association between past history and HPE findings was not statistically significant (χ² = 5.556, p = 0.475).

Association Between HPE and p57 Expression

All CHM cases (100%, n = 12) showed p57 expression in the 10-15% range. In contrast, PHM cases (n = 3) exhibited moderate expression, with 66.7% falling in the 15-30% range and the remaining 33.3% in the 30-50% range. Among POC cases (n = 9), p57 expression was more widely distributed: 11.1% showed 30-50%, 55.6% had 50-70%, and 33.4% had greater than 70% p57 positivity. Statistical analysis revealed a highly significant association between HPE diagnosis and p57 expression levels (χ² = 46.926, p < 0.001) (Table 2).

**Table 2:** Association between histopathology reports and p57 IHC CHM, complete hydatidiform mole; IHC, immunohistochemistry; PHM, partial hydatidiform mole; POC, products of conception

Histopathology report	p57 % staining of villous cytotrophoblast					Total	X²	p-value
	10-15%	15-30%	30-50%	50-70%	>70%			
CHM	12 (100%)	0 (0%)	0 (0%)	0 (0%)	0 (0%)	12	46.926	<0.001
Hydropic POC	0 (0%)	0 (0%)	0 (0%)	1	0 (0%)	1		
PHM	0 (0%)	2 (66.7%)	1 (33.3%)	0 (0%)	0 (0%)	3		
PHM	0 (0%)	0 (0%)	1 (11.1%)	5 (55.6%)	3 (33.4%)	9		

In this study, p57 IHC was performed on all cases to assist in classifying molar and non-molar gestational products. The observed staining patterns were grouped into four distinct categories: positive (>15%), negative (<10%), discordant, and divergent, each providing critical insight into the underlying genetic composition of the villous tissue.

**Figure 2:**
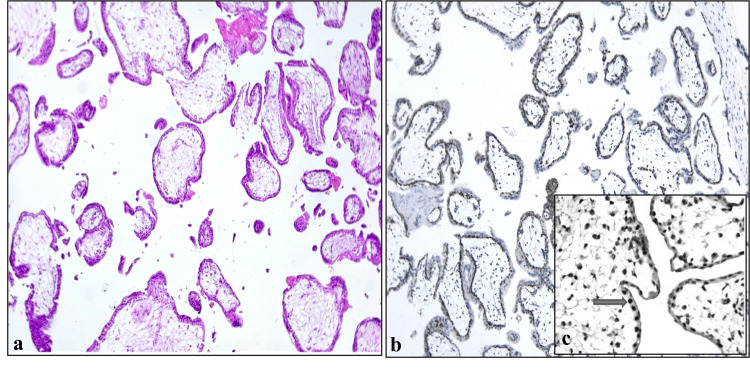
A case of normal POC showing (a) chorionic villi (IHC, ×40); (b) p57 positivity in villous cytotrophoblast and stroma (IHC, ×40); (c) inset showing p57 positivity in cytotrophoblast POC, products of conception

Positive p57 expression was characterized by strong nuclear staining in both villous cytotrophoblasts and stromal cells. This pattern was consistently observed in all cases of PHM and POC without evidence of molar pathology (Figure 2). The expression reflects the presence of maternally derived genetic material, particularly the maternal allele of the CDKN1C gene located on chromosome 11p15.5, confirming a biparental origin in these cases.

**Figure 3:**
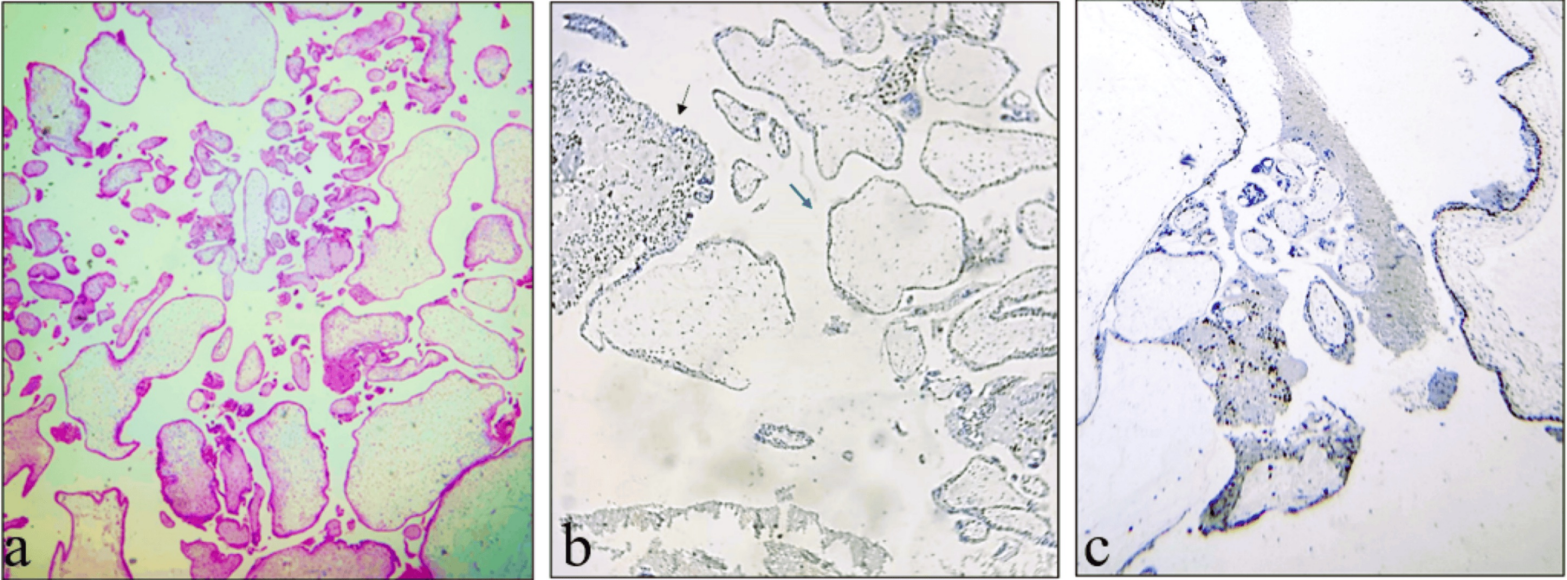
A case of complete mole showing (a) villous edema with cistern formation (H&E, ×10); (b) p57 negativity in villous cytotrophoblast (blue arrows) with positivity in extravillous sites (black arrow; IHC, ×40); (c) p57 showing discordant staining (IHC, ×40)

Negative p57 expression, defined by the complete absence of nuclear staining in both cytotrophoblasts and villous stromal cells (with appropriate positive control), was observed in the majority of CHM cases (n = 10) (Figure 3). This finding aligns with the classical androgenetic diploid genotype of CHM, where the genome is entirely of paternal origin, leading to the absence of maternally expressed p57 protein.

Discordant staining was observed in two cases of CHM, where focal or patchy p57 expression in villous cytotrophoblast and variable stromal positivity was noted (Figure 3). Discordant staining is often linked to technical artifacts, such as delayed fixation or degenerative tissue changes. Although these cases were morphologically compatible with CHM, the inconsistent staining pattern required correlation with histology and, if available, molecular analysis for confirmation.

**Figure 4:**
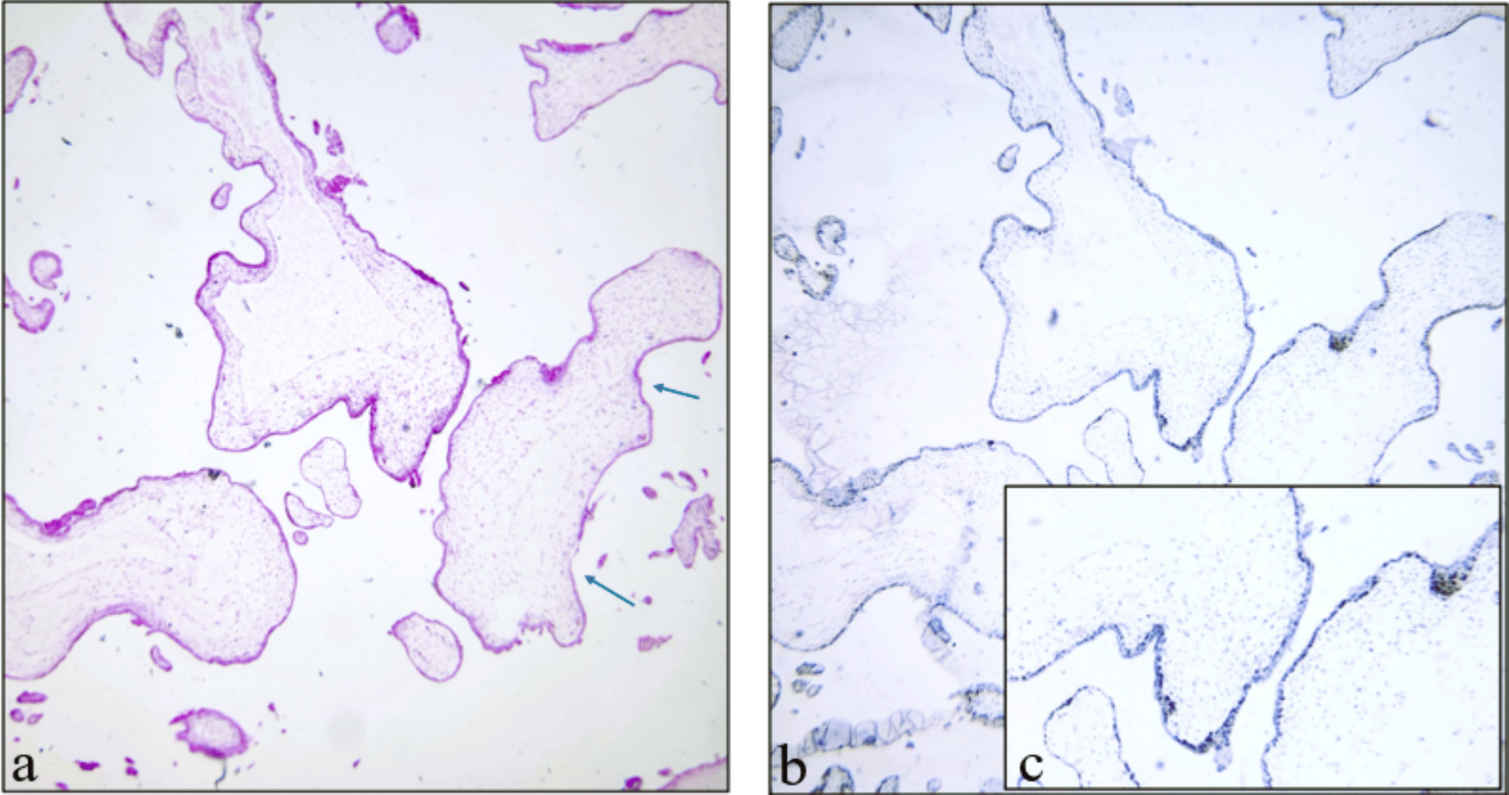
A case of partial mole showing (a) villous edema with scalloping (blue arrows) (H&E, ×40); (b) p57 positivity in villous cytotrophoblast (IHC, ×40); (c) inset image shows divergent staining

Divergent staining patterns were noted in one case of PHM. Divergent staining is defined as p57 positivity in the stromal villous component and negativity in villous cytotrophoblast (Figure 4). These findings may indicate mosaicism, partial loss of imprinting, or technical/interpretative variability. Such cases require cautious interpretation and, when possible, molecular genotyping for a definitive diagnosis.

Overall, the p57 immunostaining results showed a strong correlation with histopathological evaluation and provided vital information for differentiating molar from non-molar gestations. The presence of divergent and discordant staining patterns in a minority of cases highlights the importance of cautious interpretation and the potential need for ancillary diagnostic techniques, such as molecular genotyping, in ambiguous scenarios.

## Discussion

Molar gestations encompass a range of trophoblastic disorders that require precise diagnosis for effective clinical management, particularly to assess the risk of persistent GTD. Histopathological evaluation remains the cornerstone of diagnosis, but overlapping features between CHM, PHM, and POC, including those with hydropic changes, often result in diagnostic ambiguity. This is especially true in early gestations or poorly preserved specimens [8-10]. As a result, there has been an increasing use of ancillary techniques, particularly p57 IHC, to improve diagnostic accuracy.

Pattern of p57 IHC expression in molar gestations

p57 is a cyclin-dependent kinase inhibitor encoded by a paternally imprinted and maternally expressed gene. It is typically expressed in villous cytotrophoblasts and stromal cells when maternal genetic material is present, making its absence a highly specific marker for androgenetic diploid CHM [11,12].

In our study, p57 IHC demonstrated distinct staining patterns across the diagnostic categories. All PHM and POC cases showed strong nuclear staining in both cytotrophoblasts and villous stromal cells, which is consistent with biparental or maternal inheritance patterns. In contrast, CHM cases uniformly exhibited the complete absence of staining, aligning with their androgenetic origin. These findings are consistent with previous studies by Castrillon et al. and Zhao et al., who reported the high specificity of p57 negativity for CHM diagnosis [11,13].

Interestingly, we observed two unique staining patterns: divergent and discordant staining. Divergent staining was identified in one case of PHM, with positivity in the stromal villous component and negativity in the villous cytotrophoblast. Divergent staining may arise from technical factors, tissue preservation issues, or biological variability, such as confined mosaicism or partial maternal imprinting [14,15]. Similar findings were described by Carreon and Roberts, who observed inverted or mosaic p57 staining in third-trimester placentas, emphasizing the need for caution when interpreting isolated staining patterns [16].

Discordant staining, noted in two CHM cases in our study, presented as patchy, weak staining or staining without proper internal control validation. Gaillot-Durand et al. addressed this phenomenon, analyzing 70 cases of hydropic POC and reporting discordant patterns in a subset. They advised careful correlation with clinical and histological findings to avoid misdiagnosis [17].

Effectiveness of p57 IHC in the diagnosis of molar gestation

Our study underscores the significant diagnostic utility of p57 IHC, especially in identifying CHM. The absence of p57 expression in both cytotrophoblasts and stromal cells serves as a clear indicator of androgenetic origin, a hallmark feature of CHM. In this context, our findings support the conclusions of larger prospective studies by Banet et al. and Vang et al., who demonstrated that p57 IHC, when combined with morphology and genotyping, significantly improves diagnostic reproducibility [18,19].

Of particular note, Vang et al. highlighted that even experienced pathologists can encounter diagnostic discordance when relying solely on morphology, particularly when distinguishing PHM from HA, emphasizing the critical role of ancillary techniques [19]. Our study supports this perspective: despite morphologic overlap, p57 IHC allowed for clear classification in most cases, including those with morphologically ambiguous specimens.

However, p57 IHC does have limitations. Technical factors, such as suboptimal fixation, delayed processing, and section artifacts, can lead to equivocal or misleading staining. Moreover, rare genetic conditions, including biparental CHM or androgenetic/biparental mosaicism, can result in atypical p57 expression [20,21]. In such cases, molecular genotyping remains the gold standard for a definitive diagnosis, as demonstrated in studies by Ronnett et al. and Murphy et al., who advocate for an algorithmic diagnostic approach that integrates morphology, IHC, and genotyping [20,21].

Additionally, the importance of internal positive controls, such as staining of intermediate trophoblasts or maternal decidua, cannot be overstated. p57 staining should always be interpreted in the context of these controls, as false negatives can occur in the absence of staining in internal control elements.

Giacometti et al. emphasized this in a recent review, suggesting algorithmic approaches for challenging cases, particularly when tissue preservation is poor or patient history is limited [22]. Their recommendations reinforce the need for a multidisciplinary approach, combining clinical data, pathology, IHC, and molecular studies, to ensure optimal diagnostic accuracy.

From slide to signature: reporting p57 and HPE

When signing off on reports for HPE and p57 IHC, accuracy and clarity are paramount. Pathologists must integrate histomorphological findings with p57 staining results to ensure a definitive diagnosis. p57 serves as a reliable marker for distinguishing complete moles from partial moles and hydropic abortions. Proper interpretation of nuclear staining patterns in cytotrophoblasts and stromal cells is essential for accurate classification.

A consistent and well-documented reporting format fosters diagnostic confidence and informs clinical decisions. Reports should include details on tissue adequacy, staining quality, and the interpretation of results in the context of clinical data. In cases with discordant findings, careful phrasing is crucial, and molecular confirmation may be needed for clarification. Pathologists must remain aware of the diagnostic limitations of p57 IHC alone.

Clear conclusions and recommendations enhance the clinical utility of the report, ultimately contributing to better patient care and improved interdisciplinary communication.

Limitations

This study has a few limitations, mainly due to the rarity of the cases. As a single-center study, there is the potential for selection bias. Additionally, the interpretation of p57 IHC is partly subjective, and interobserver variability may affect diagnostic consistency. Finally, the absence of long-term clinical follow-up limits the ability to assess the prognostic value of p57 expression.

## Conclusions

This prospective study demonstrates that p57 IHC is a valuable diagnostic tool for the accurate identification of molar pregnancies. The p57 staining patterns observed in molar gestations were distinct, with clear differential expression between complete and partial molar pregnancies, as evidenced by a statistically significant p-value. The positive results of this study highlight the potential utility of p57 IHC in enhancing diagnostic precision, reducing the risk of misdiagnosis, improving report signing efficiency, and guiding appropriate management. Future studies with larger, multicenter cohorts and integrated molecular analysis are recommended to further assess risk stratification and prognostication.
